# Nanobubble-Assisted Flotation of Apatite Tailings: Insights on Beneficiation Options

**DOI:** 10.1021/acsomega.1c01551

**Published:** 2021-05-19

**Authors:** Vitalis Chipakwe, Rickard Jolsterå, Saeed Chehreh Chelgani

**Affiliations:** † Minerals and Metallurgical Engineering, Dept. of Civil, Environmental and Natural Resources Engineering, Luleå University of Technology, SE-971 87 Luleå, Sweden; ‡ LKAB Research & Development, SE-971 28 Luleå, Sweden

## Abstract

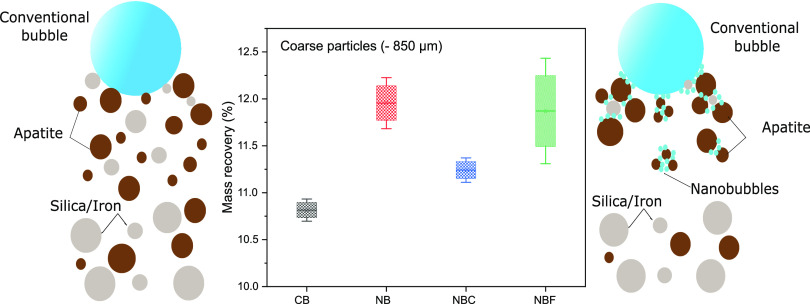

Processing of materials that originated from tailings of industrial plants (with a wide range of particle size distribution, “PSD”) without grinding has several advantages since mines are faced with a lot of pressure to minimize their environmental impacts. This article indicates that the introduction of submicron bubbles (known as nanobubbles, “NBs”) to conventional flotation could improve the separation efficiency of valuable minerals from their associated gangue phases. It was demonstrated that metallurgical responses (recovery, grade, selectivity, and kinetics) of NB flotation could improve compared to those of conventional tests. Various hydrodynamic cavitation setups for NB generation may lead to different metallurgical responses. In general, the addition of surfactants (frothers and collectors) for NB generation could increase both mass and water recoveries, which would be key factors on selectivity. Selectivity is also markedly dependent on the PSD of feed, and the selectivity of NB flotation is improved significantly by decreasing the feed size. In general, generation of NBs in the presence of a frother leads to higher flotation metallurgical responses than in the presence of a collector.

## Introduction

Beneficiation of nonconventional sources and waste streams has gained popularity due to the current demand for minerals and exhaustion of primary sources. As such, apatite ((Ca_5_(PO_4_)_3_)(F, Cl, or OH)) in apatite-iron tailings has become an essential source of phosphates for agricultural purposes. Tailings repositories pose an environmental concern^1−3^ and, in some cases, present reprocessing opportunities.^3,4^ Due to the stricter environmental regulation, mining houses are under pressure to ensure the impacts are mitigated. On the other hand, tailings’ reprocessing as secondary resources often bring economic advantages as grinding is usually minimal or not required. With the fast-paced developments of more efficient machines and improved technologies, mining houses are considering reprocessing these tailings to maximize profit maximization, reducing tailings dam’s issues.

In pursuit of their continued efforts in reducing environmental impact and sourcing of critical raw materials, Luossavaara-Kiirunavaara Aktiebolag (LKAB) has embarked on a tailings retreatment project. A large apatite-iron tailings deposit is located north of Sweden in Kiruna and Malmberget, containing a substantial amount of P_2_O_5_ (4–8%) from the previous magnetite recovery. Efforts are being made to ensure the efficient extraction of the minerals of interest with a minimum CO_2_ footprint. The possibility of coarse flotation is being investigated given the relatively liberated mineral particles from the mineralogy.^5^ Coarse flotation (an extensive particle size distribution, PSD) presents an attractive beneficiation option to eliminate the energy-intensive grinding. This process also reduces water usage and reagent consumption and makes the tailings dam more stable due to the deposition of coarser materials. However, conventional mineral separation by flotation has poor performance on coarse particles (>150 μm).

For addressing these challenges, the application of submicron bubbles in the flotation (known as “NB flotation”) of pure phosphate minerals, and some on actual ores, has been understudied for over a decade (Table 1). The typical decrease in coarse particles’ recovery is often attributed to detachment.^6,7^ For promoting attachment, the use of nanobubbles has been reported to be effective,^8−11^ mainly due to their high stability, great longevity, and rapid attachment to hydrophobic surfaces.^12^ Studies have been conducted to assess the influence of bubble sizes generated in various hydrodynamic cavitation systems.^8,13,14^ Additionally, liquid properties have been addressed, namely, temperature, surfactant type, and dosage. Through the NB generation, an increase in the surfactant concentration has been found to reduce bubble sizes and improve their stability.^14−17^ The change in surface tension explains this as the liquid’s surface tension decreases, and the bubbles formed become finer.^13,14^ Many NB flotation studies reported improving flotation recoveries, grades, and kinetics and even reduced reagent consumption.^10,18−23^ In a recent study, Pourkarimi et al.^24^ indicated that generation of NBs in the presence of selective collectors could significantly increase the flotation efficiency of fine phosphate ore samples. Surprisingly, surface analyses of flotation concentrates indicated that the amounts of flotation collectors adsorbed onto the surface of floated particles were lower in the presence of NBs than in their absence. However, adding nanobubbles to a conventional flotation system can also increase the entrainment rate. Entrainment could be the main reason for improving the metallurgical responses of pure mineral flotation, where a selective separation would not be an issue.^25^

**Table 1:** Summary of Existing Application of Nanobubbles on Phosphates

material	NB generation	particle size range	observed change (P_2_O_5_)	references
phosphate ore	F-507 (glycol frother), FA-18G (fatty acid)	–1180 to +150 μm	+10–30% recovery, +1–1.9% grade	(10)
apatitic ore		–75 μm	+8.9% recovery, −1.9% grade	(21)
phosphate ore	Flo-Y-S	–38 μm	+35.4% recovery, +3.4% grade	(26)
pure apatite		–75 μm	+35% mass recovery	(21)

This study will examine the effect of nanobubbles generated with/without surfactants on the selective flotation performance of coarse apatite from Malmberget tailings (Sweden). For the first time, this investigation assessed the effect of entrainment through NB flotation of coarse particles originated from sorting plant tailings. In general, NB flotation has been recommended for fine particles; therefore, for comparison purposes, two size fractions of the samples were subject for NB flotation. The overall goal is assessing the reprocessing of tailings as received (−850 μm) or sieved size fractions (−106 μm, typical feed size for a flotation separation) in flowsheet development under different conditions using NBs generated by various reagents.

## Results and Discussion

### Metallurgical Responses

**Figure 1:**
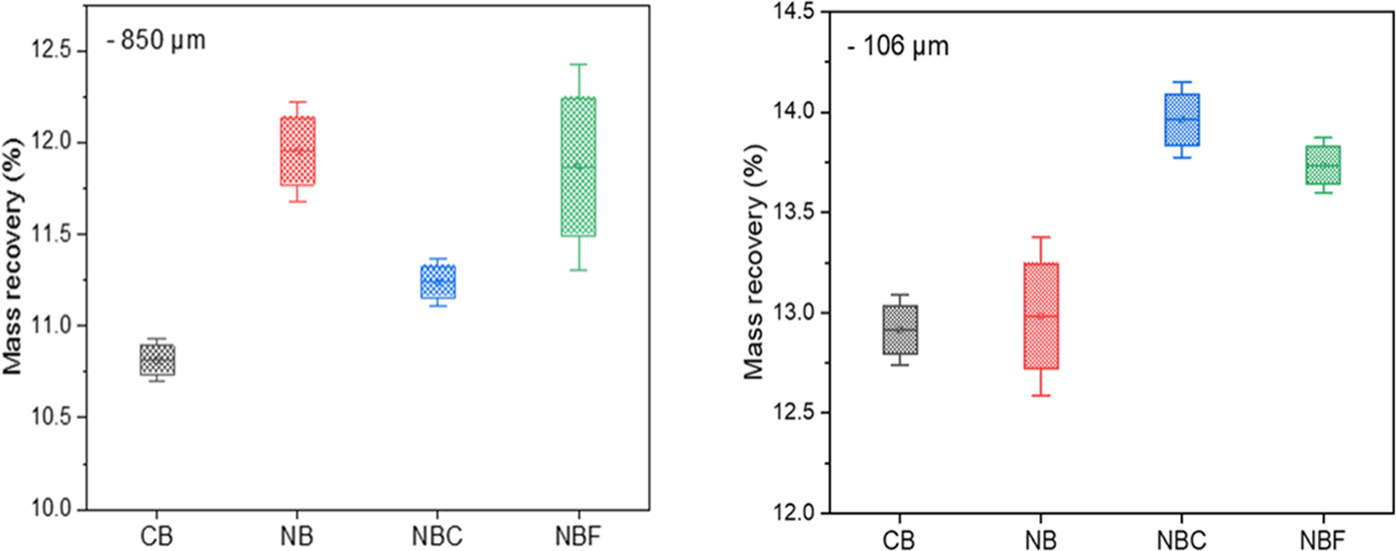
Apatite flotation mass recovery for various tailings.

**Figure 2:**
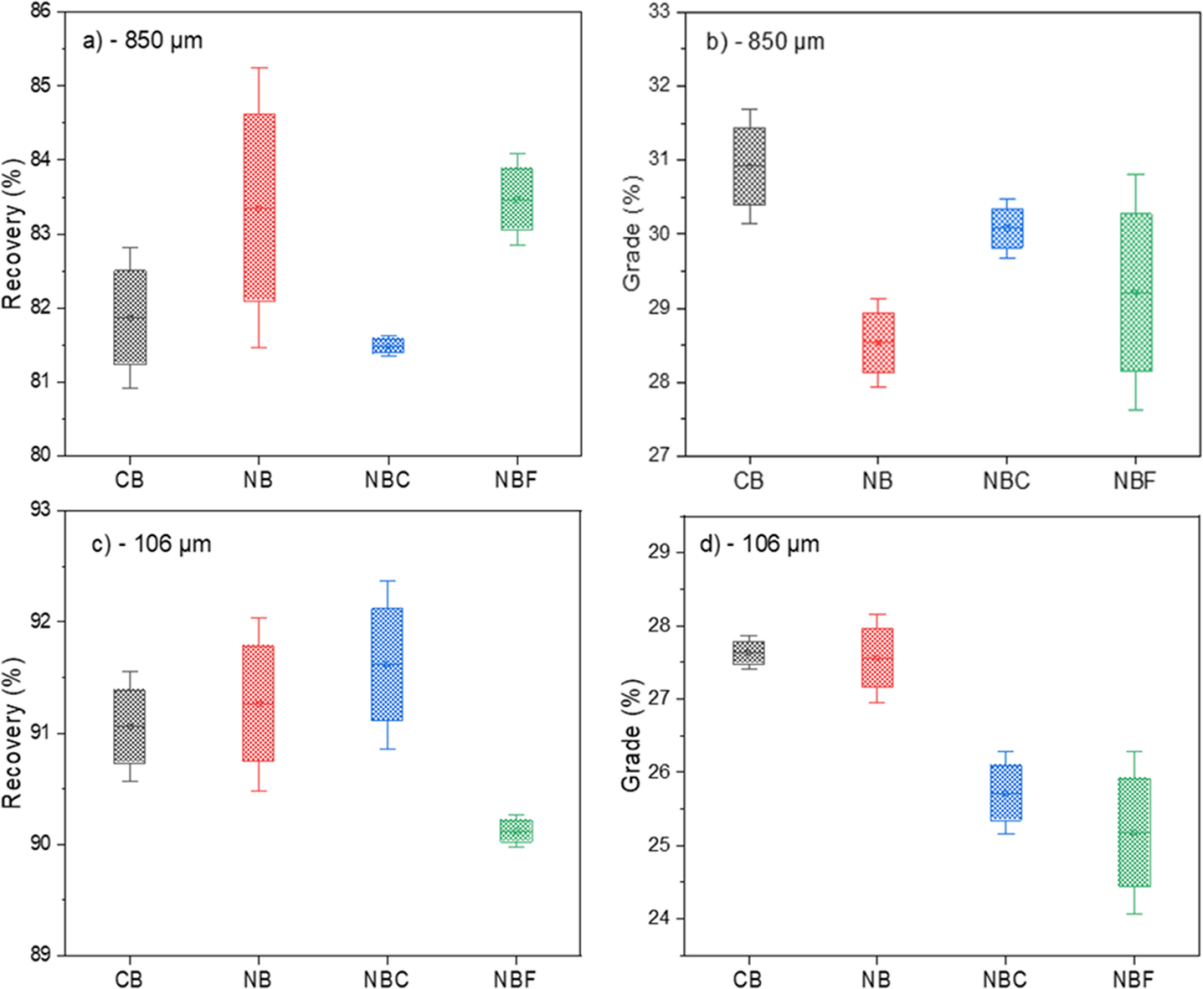
Variation of P_2_O_5_ recovery and grade for (a, b) −850 and (c, d) −106 μm fractions.

**Figure 3:**
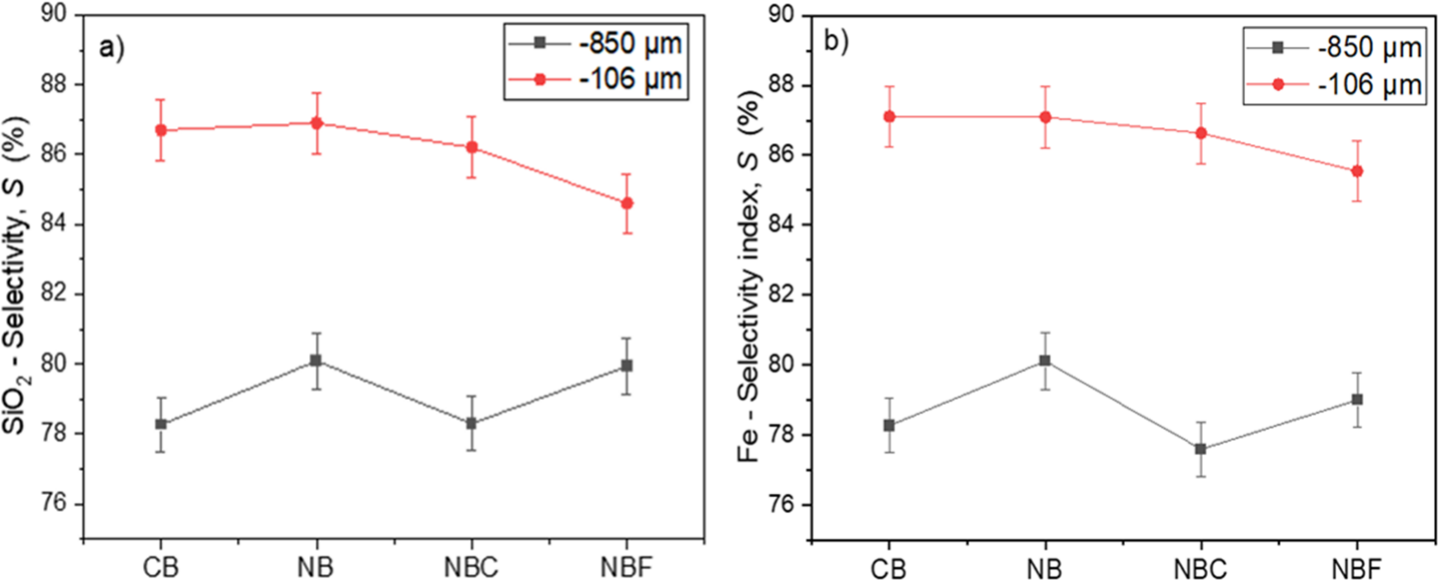
Flotation selectivity based on (a) silica recovery and (b) iron recovery under different conditions and particle size fractions.

Flotation test results (Figure 1) illustrated that the mass recovery in the presence of all NB flotation setups (NB—nanobubbles, NBC—nanobubbles with collector, NBF—nanobubbles with frother) was higher than that in the conventional test (CB). NB and NBC experiments showed the highest mass recoveries for −850 and −106 μm, respectively (on average). Although the NB setup (without reagent addition for the NB generation) provided the highest P_2_O_5_ recovery, it resulted in the lowest P_2_O_5_ grade compared to other experiments for the as-received samples (Figure 2a,b). For −106 μm samples, NB and NBC indicated the highest P_2_O_5_ recovery and grade, respectively (Figure 2c,d). These differences clearly demonstrated the effect of particle size on a selective NB flotation separation. This critical point could not be addressed by considering pure minerals for a process assessment. Flotation outcomes revealed that the NB setup for both size fractions could lead to a high selectivity index (Figure 3). It should be noted that despite high recoveries reported in all NB-assisted setups, the grade was marginally lower than that in the CB for both size fractions. In general, metallurgical responses for the smaller particle size (−106 μm) were higher than those for the coarser one (−850 μm). The introduction of NBs to the coarser feed size (−850 μm) showed a pronounced effect compared to the fine feed size (−106 μm). This could be attributed to the relatively high proportion of the appropriate particle size fraction for the optimum flotation performance (+38–150 μm).^10,27^ The lower grades could be attributed to fine gangue minerals’ entrainment by water and the entrapment effect due to the frother. Lei et al.^25^ showed that NBs’ introduction increased the entrainment rate during the flotation of kaolinite particles, which correlated with increased water recovery. Pourkarimi et al.^28^ reported increasing entrainment when adding a frother during the NB generation through the apatite flotation.

### Flotation Kinetics

**Figure 4:**
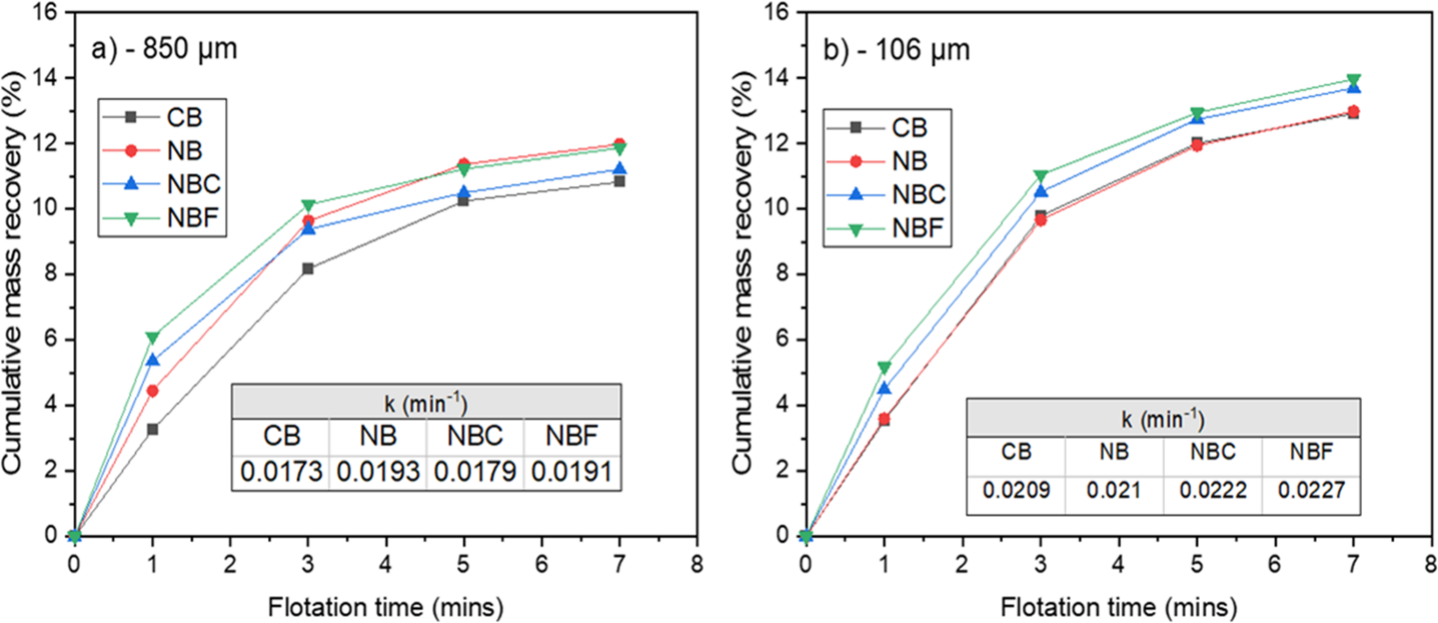
(a, b) Cumulative mass as a function of flotation time.

The mass recovery–time profile indicated that NBF could result in the fastest flotation kinetics and highest overall recovery (Figure 4). In general, all NB setups for both the examined size fractions resulted in a higher *k* than CB. After 1 min, the use of NBF doubled the recovery (6.1%) compared to the conventional bubbles (3.3%) for the −850 μm size fraction. After 7 min, NB, NBC, and NBF had the highest mass recoveries of 12.0, 11.2, and 11.9%, respectively, compared to 10.6% for the conventional bubbles for the −850 μm samples. *k* for the finer size fraction was higher than that for the as-received feed. The fact that the curves for NB, NBC, and NBF were always above the conventional bubbles’ curves indicated the improved flotation *k*. This is consistent with the findings reported in the literature where nanobubbles result in an increased flotation rate constant.^18−22^ From the classical first-order model used (eq 1), the correlation coefficients, *R*^2^, were 0.9958, 0.9989, 0.9853, and 0.9988 for CB, NB, NBC, and NBF, respectively for the −850 μm samples. Whilst the −106 μm samples had *R*^2^ values of 0.9925, 0.9968, 0.9879, and 0.9818 for CB, NB, NBC, and NBF, respectively. The high *R*^2^ values showed good agreement between the experimental and calculated values.

**Figure 5:**
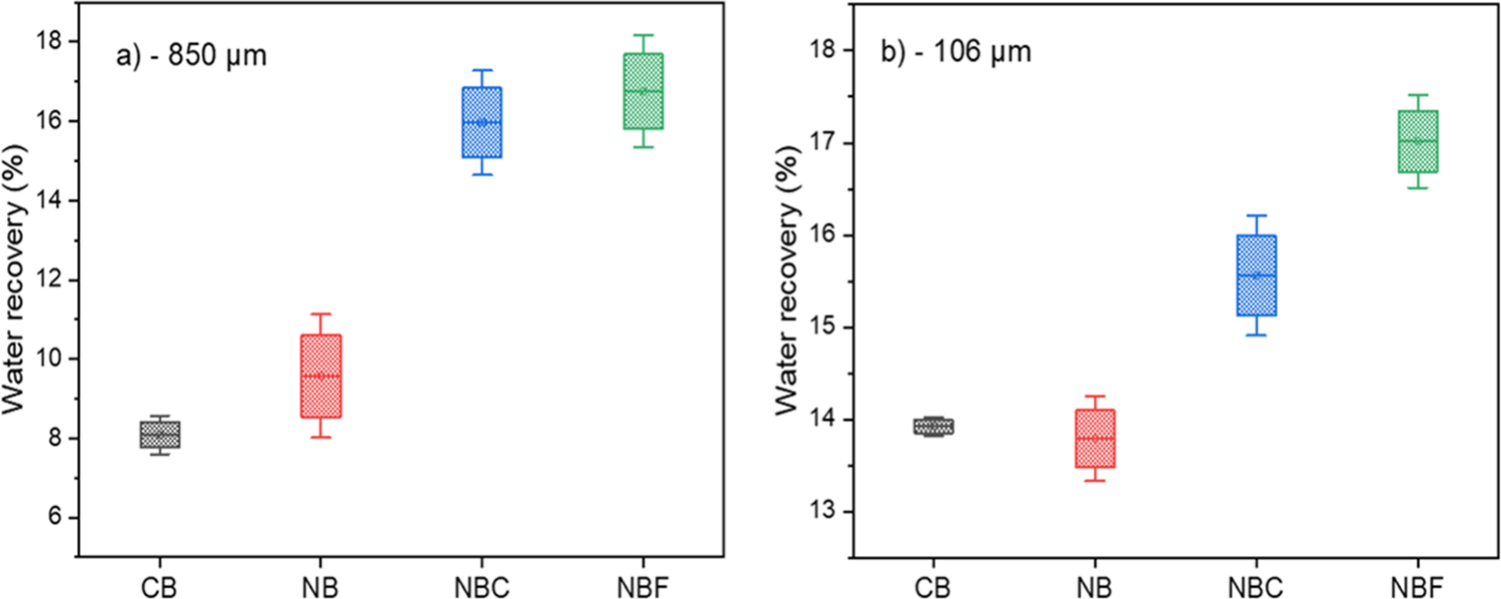
(a, b) Cumulative water recovery for various flotation setups.

**Figure 6:**
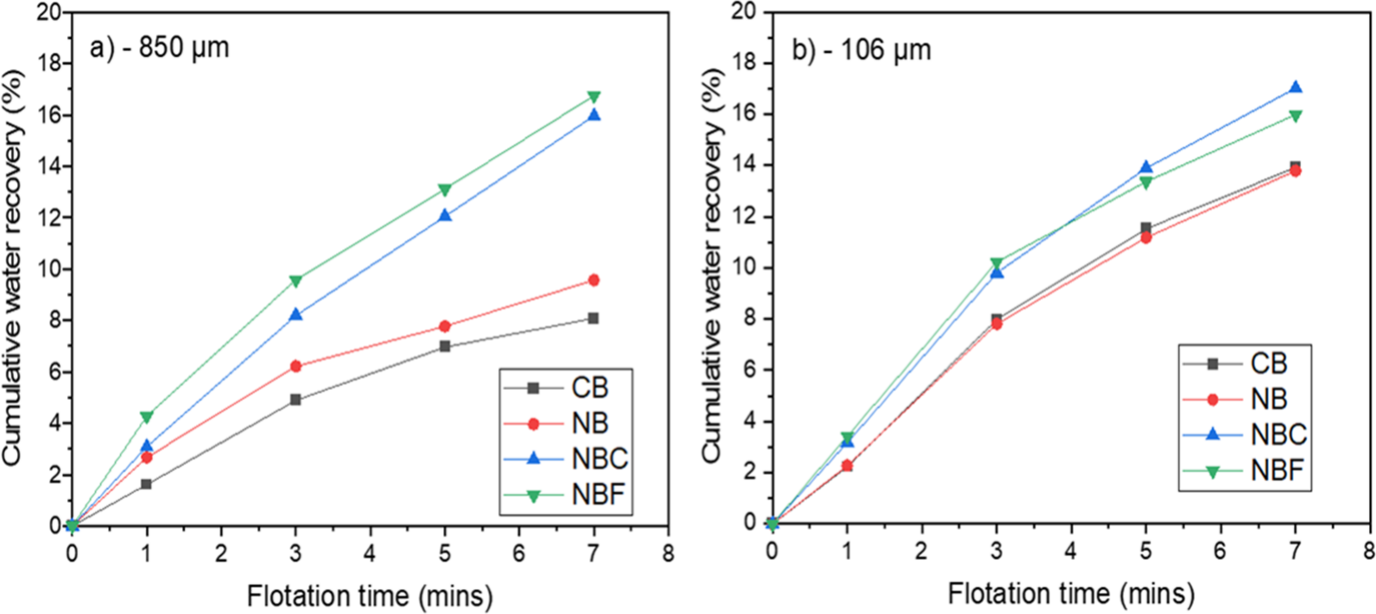
(a, b) Cumulative water recovery as a function of flotation time.

The water recovery reported in Figure 5 is the fraction of water that recovered in the concentrate (including initial water in the cell and water added to maintain the pulp level). It can be seen that for the NBC and NBF, the water recovery doubled when compared to CB for the −850 μm samples. The CB had a consistently lower water recovery for both size fractions (Figure 6).

In other words, it could be observed that the predicted *k* values in the presence of NBs (all setups) are high compared to those of CB (Figure 5). NBF had the highest *k* values in both size fractions. As expected, the flotation rate constant for the coarser (−850 μm) fraction is lower compared to that for the fine (−106 μm) fraction, consistent with the findings by Nazari and Hassanzadeh.^8^ In their investigation on the effects of different surfactants in the bulk generation of nanobubbles, they reported the following order of the effect on the rate constant: dodecylamine > pine oil > MIBC (alcohol) > A65 (polypropylene glycol). The flotation rate, *k*, as expected, also decreased as the feed particle size increased regardless of the surfactant used. The pronounced effect on the fine fraction, when compared to the coarser fraction, could be attributed to the relatively high proportion of the appropriate particle size fraction for optimum flotation performance (+38–106 μm).^10,27^ However, comparing both fractions shows the following change when comparing conventional bubbles and nanobubbles: the −850 μm sample has the biggest change compared to the −106 μm one.

### Entrainment

**Figure 7:**
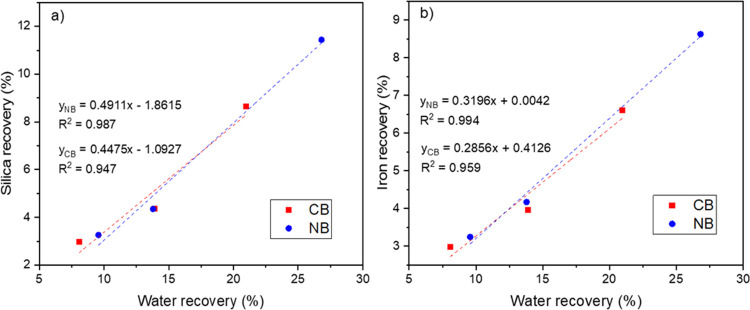
(a) Silica and (b) iron recoveries vs cumulative water recovery for conventional bubbles and nanobubbles.

Table 2 shows the grades of P_2_O_5_, P, Fe, and SiO_2_ for both size fractions under different hydrodynamic conditions. The grades of the gangue phases (Fe and SiO_2_) slightly increased with reagents’ introduction (NBC and NBF). This increase, to some extent, was higher for the −106 μm sample than the −850 μm one. The increase in gangue materials (SiO_2_ and Fe) was accompanied by a slight decrease in P_2_O_5_, which means that a higher water recovery resulted in a concentrate grade reduction. To further confirm this, analysis shows a strong linear correlation between both SiO_2_ and Fe recoveries and water recovery (Figure 7). The observed linear relationship is not unique and has been reported in other investigations.^29,30^ Noticeably, the slope for NBs is steeper, which approximates the degree of entrainment from (*R*_ENT_ = ENT · *R*_w_)^29^ compared to conventional bubbles supporting the linear relationship.

**Table 2:** Comparison of Concentrate Grades under Different Hydrodynamic Conditions

	–850 μm				–106 μm			
content (%)	P_2_O_5_	P	Fe	SiO_2_	P_2_O_5_	P	Fe	SiO_2_
CB	31.4	13.7	3.6	12.4	27.6	12.1	4.7	15.0
NB	28.9	12.6	3.5	12.3	27.6	12.0	4.9	14.9
NBC	30.1	13.1	3.9	12.7	25.7	11.2	5.0	17.2
NBF	30.3	12.8	4.5	13.3	25.2	11.0	4.7	18.2

The degree of entrainment is more pronounced in the scenario where nanobubbles were generated with surfactants. The hydrophilic particles that get entrained by the nanobubbles to the froth zone evident from the increased water recovery remain entrapped in the stabilized froth (due to the surfactant), thus reporting to the concentrate. Therefore, the increased entrainment (from the high water recovery and small particle size, low inertia) and entrapment in the froth zone due to the stable froth resulted in a lower concentrate grade. The addition of surfactant stabilizes the bubbles but also plays a critical role in the selective drainage of water in the froth zone.^31^ Kracht et al.^30^ investigated the effect of the surfactant type and concentration on the entrainment rate. The results show that the entrainment rate depends on the reagent type and indicates that it is higher for MIBC (alcohol) than for Atrac 1563 (carboxylic acid). Previously, it was reported that alcohols tended to increase entrainment compared to polyethylene glycols.^32^ Of the alcohols investigated, MIBC was found to give more entrainment compared to hexanol despite similar molecular weights, and the difference was attributed to the branched structure of MIBC.^30^ This could also explain the increased entrainment for nanobubbles generated with MIBC compared to Atrac 1563, a carboxylic acid.

## Conclusions

The application of nanobubbles through flotation of coarse apatite tailings evidently improved the process’ metallurgical responses compared to flotation by conventional bubbles only. The results indicated that nanobubble flotation of the as-received apatite tailings (−850 μm) was an attractive option that can minimize the CO_2_ footprint of mineral processing by eliminating regrinding. The nanobubbles generated with surfactants (collectors and frothers) could cause a decrease in grade, mainly because of the increased entrainment rate. For maximum benefit of the nanobubbles, it could be proposed that the nanobubbles generated with different surfactants (frothers or collectors) should be used in the different flotation stages (rougher or cleaner). Where recovery is more preferred than grade control, adding reagents could be beneficial, and for the cleaner stages, the use of surfactants in the generation stage is not recommended. Further work is required to optimize the hydrodynamic conditions and surfactant types to reduce the amount of water recovery associated with nanobubbles, which will reduce the rate of entrainment.

## Materials and Methods

**Figure 8:**
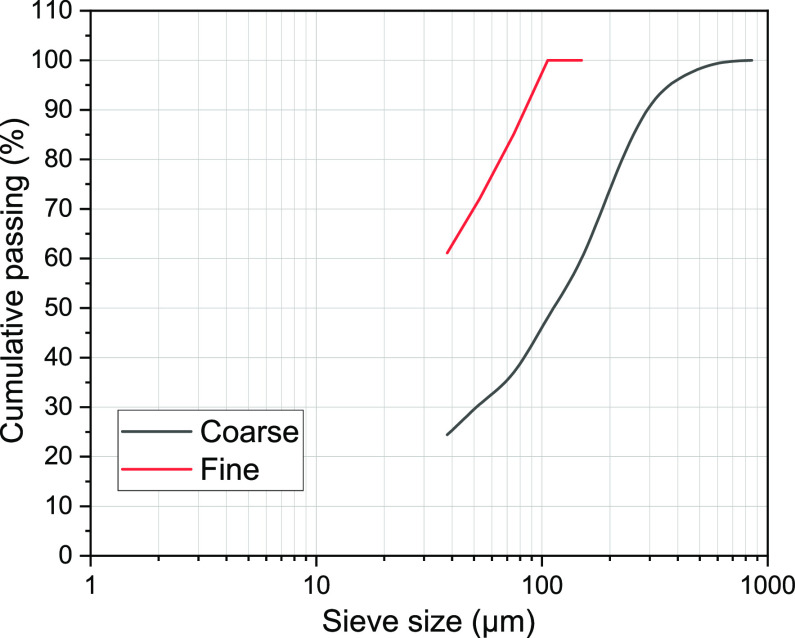
Particle size distribution of the two fractions used for flotation separation.

For this study, the tailings samples were received from LKAB’s Malmberget mine with the chemical analysis from X-ray fluorescence (XRF) presented in Table 3. The Kiruna/Malmberget-type apatite’s mineralogy is mainly fluorapatite and some chlorapatite, which occurred interstitially to magnetite with grains mostly between 0.05 and 1 mm.^5^ The as-received material was relatively coarse with *F*_80_ = 227 μm (coarse), which was sieved to give a −106 μm fraction (fine) with *F*_80_ = 65 μm for further assessments (Figure 8). The two fractions represent two beneficiation options: (1) −850 μm, the material in its current state on the sorting plant tailings forming the flotation feed directly and (2) −106 μm, the material after classification as the flotation feed with the coarser fraction going for more grinding first.

**Table 3:** Chemical Analysis of the Tailings Sample

content (%)	Fe	P	SiO_2_	K_2_O	Al_2_O_3_	CaO	MgO	MnO	TiO_2_	V_2_O_5_	P_2_O_5_
as-received	12.9	1.81	45.1	2.7	9.5	8.9	5.4	0.04	0.68	0.05	4.15
after sieving	15.4	1.7	44.5	2.7	9.7	8.7	4.9	0.05	0.78	0.05	3.92

### NB Generation Setup

The nanobubbles were produced using an air-in-water microdispersion generator developed by Living Energies & Co. (Japan). The NB generation is carried out based on the cavitation through a Venturi tube under a working pressure of ∼2 MPa. The process is controlled by the feed inlet valve, pump speed, air uptake valve, and operating pressure. For the NB generation, two surfactants were used: (1) Atrac 1563, a fatty acid collector with frothing properties, “NBC”, and (2) methyl isobutyl carbinol (MIBC), an alcohol-based frother, “NBF” (molecular weight: 102), at a dosage of 20 ppm based on a work reported elsewhere by Nazari and Hassanzadeh.^8^ The NBs were delivered to the flotation cell as a concentrated air dispersion at mild-pulp agitation before the cell air valve was opened for the flotation process. The NBs were introduced in varying quantities for a set time to ensure a constant supply throughout the flotation time. The typical size range of NBs generated is in a range of 100–200 nm.^33^

### Flotation Tests

All the flotation experiments were conducted in the rougher stage. For both conventional bubble size (CB) flotation and different NB flotation setups (NB (without reagents), NBF, and NBC), the respective feed material (both fractions of apatite tailings samples, separately) was pulped with Luleå tap water to give 35 wt % solids. The pulp was conditioned for 5 min in an automated Outotec GTK LabCell, a laboratory-scale mechanical cell (Table 4). The pH was adjusted to 9.0 using 5 wt % sodium hydroxide solution recommended by the collector supplier (Nouryon) and as reported elsewhere by Potapova et al.^34^ The pulp was conditioned for 1 min after which 500 g/t water glass was added as a depressant. Atrac 1563 (200 g/t) was added to the pulp and conditioned for 2 min. No frother was added to the flotation cell since Atrac 1563 has frothing properties itself. Once the air was opened, the concentrates were collected at 1, 3, 5, and 7 min. At the end of the flotation process (after 7 min), the respective samples and the remaining material reported to the tailings were collected in different containers. The flotation products were weighed and dried at 110 ° C. After drying, the concentrates were stored and packed in plastic bags for further PSD and chemical analyses by XRF to determine metallurgical responses.

**Table 4:** Operation Variables for the Flotation Cell

parameter	value
cell volume	2000 cm^3^ with a 45 mm rotor diameter
impeller speed	1300 rpm
airflow rate	2 L/min
solid content	35 wt %

To further evaluate the NBs’ effect on flotation kinetics, the flotation rate constant *k*, for each scenario, was calculated using the first-order model (eq 1)
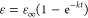
1

The separation efficiency can be expressed by the selectivity index, *S*, which is given by eq 2
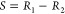
2where *R*_1_ is the recovery of P_2_O_5_ and *R*_2_ is the recovery of SiO_2_/Fe. A higher selectivity index *S* extrapolates better selectivity for the process.^19^ All experiments were repeated twice, and their averages were reported. Accordingly, experimental errors were determined at a 95% confidence level.
